# HIV risk behaviours among injecting drug users in Northeast India following scale-up of a targeted HIV prevention programme

**DOI:** 10.1186/1471-2458-11-S6-S9

**Published:** 2011-12-29

**Authors:** Gregory Armstrong, Chumben Humtsoe, Michelle Kermode

**Affiliations:** 1 Nossal Institute for Global Health, University of Melbourne, Victoria, Australia; 2 Project ORCHID, Emmanuel Hospital Association, Guwahati, India

## Abstract

**Background:**

In the Northeast Indian states of Manipur and Nagaland there has been an ongoing HIV epidemic among injecting drug users (IDUs) since the mid-1990s. Project ORCHID is an Avahan-funded HIV prevention project that has been working in selected districts of Manipur and Nagaland since 2004. It supports local partner non-government organisations (NGOs) to deliver a range of harm reduction interventions, and currently reaches approximately 14,500 IDUs across the two states. To assess changes in HIV risk behaviours two Behavioural Tracking Surveys (BTS) were undertaken among IDUs in 2007 and 2009.

**Methods:**

The BTS used respondent driven sampling (RDS) to recruit adult male IDUs (18 years of age and above) from Ukhrul and Chandel districts in Manipur, and Kiphire and Zunheboto districts in Nagaland. This paper reports on analysis of socio-demographics, drug use and injecting practices, sexual behaviour and condom use, knowledge of HIV, and exposure to interventions. Descriptive data were analysed using RDSAT, and odds ratios were calculated in SPSS.

**Results:**

The proportion of IDUs reporting NOT sharing needles / syringes at last injection increased substantially in Ukhrul (59.6% to 91.2%) and Zunheboto (45.5% to 73.8%), remained high in Chandel (97.0% to 98.9%), and remained largely unchanged in Kiphire (63.3% to 68.8%). The use of condoms with regular partners was low in all districts at both time points. In Ukhrul, Kiphire and Zunheboto the proportion of IDUs using condoms during sexual intercourse with a casual partner increased substantially to approximately 70-85%, whilst in Chandel the increase was only marginal (57.4% to 63.6%). Exposure to NGO HIV prevention interventions was significantly associated (p<0.05) with lower odds of sharing needles during the previous month (Nagaland, OR=0.63; Manipur, OR 0.35).

**Conclusion:**

Despite district-level differences, the results from this BTS study indicate that exposure to HIV prevention services, predominately delivered in this region by NGOs, is associated with a reduced likelihood of engaging in HIV risk behaviours. IDUs using HIV prevention services are more likely to engage in safe injecting and sexual practices, and effort is required to sustain / increase opportunities for IDUs to access these services. These outcomes are a noteworthy achievement in a very challenging context.

## Background

Injecting drug users (IDUs) are at high risk of infection with blood-borne viruses including HIV, hepatitis B and hepatitis C, and are increasingly being targeted by policies aimed at preventing the spread of HIV [1]. Approximately 10% of HIV infections worldwide are attributable to injecting drug use [2], and many countries in Asia, including Thailand, Myanmar, Indonesia, Bangladesh, Nepal and India are confronting serious HIV epidemics among IDUs [3].

In the Northeast Indian states of Manipur and Nagaland, approximately 2% of the population injects drugs [4], most commonly heroin and Spasmoproxyvon (SP, a synthetic opioid analgesic that contains dextropropoxyphene, dicyclomine hydrochloride and paracetamol). Consequent to sharing of injecting equipment during drug use, Manipur and Nagaland are two states that consistently report a high HIV prevalence, and in the case of Manipur, the highest in India. According to 2009 sentinel surveillance figures, HIV prevalence among the general population in Manipur was 1.4%, and in Nagaland was 0.8% [5]. Among IDUs, HIV prevalence was 29% in Manipur (2008) and 1.9% in Nagaland (2007) [6]. An Integrated Biological and Behavioural Assessment (IBBA) conducted in 2006 in Manipur and Nagaland reported comparable HIV prevalence figures (23.1% and 32.3% in Bishnupur and Churachandpur in Manipur, and 1.1% and 1.8% in Phek and Wokha in Nagaland) [7]. The prevalence of hepatitis C infection was very high in Manipur (56% in Bishnupur and 78% in Churachandpur), but lower in Nagaland (5.4% in Phek and 16.7% in Wokha). The IBBA study also found widespread sharing of injecting equipment with between 35% and 70% of IDUs reporting that they sometimes engaged in receptive sharing of needles and syringes.

Northeast India is an ethnically distinct and geographically isolated part of the country characterised by a longstanding civil insurgent struggle, poverty and unemployment. Many communities are located in geographically difficult-to-reach areas. The response to HIV and injecting drug use in this geo-politically complex environment was historically punitive and coercive, but a harm reduction approach has been government policy since the mid 1990s [8]. This currently includes provision of needle and syringe exchange programs, condom distribution, opioid substitution therapy (recently evaluated [9]), treatment for sexually transmitted infections (STIs), and behaviour change communication.

The HIV prevention response in these two states is coordinated by the government through the National AIDS Control Organization (NACO) and the respective State AIDS Control Societies (SACS). Alongside this, Avahan (Bill & Melinda Gates Foundation in India) has funded Project ORCHID to provide HIV prevention interventions in selected districts of Manipur and Nagaland over a ten year period (2004-13) [10]. Project ORCHID is a partnership of the Emmanuel Hospital Association, New Delhi, India and the Australian International Health Institute, University of Melbourne, Australia. Regardless of funding source, most of the targeted HIV prevention interventions in Manipur and Nagaland are delivered in the field by local non-government organizations (NGOs).

To assess changes in HIV risk behaviours two Behavioural Tracking Surveys (BTS) were undertaken among IDUs in 2007 and 2009. This paper reports on the comparison between baseline (2007) and follow-up (2009) BTS data obtained from IDUs in two districts of Manipur (Ukhrul and Chandel) and two districts of Nagaland (Kiphire and Zunheboto) where Project ORCHID NGOs are operational. The objectives are: 1) to quantify changes over time with respect to the characteristics of injecting drug users, HIV risk behaviours, awareness of HIV, and exposure to harm reduction interventions, and 2) to estimate associations between exposure to interventions and needle sharing, condom usage and participation in HIV testing.

## Methods

### Study design

The BTS is a cross-sectional survey that collects behavioural data via an interviewer-administered questionnaire, which was repeated at two different time points (2007 and 2009) in two districts of Manipur and two districts of Nagaland. Ethics approval for this study was obtained from the Institutional Review Board of the Emmanuel Hospital Association Research Ethics Committee, New Delhi.

### Sampling method

Respondent driven sampling (RDS) was used to recruit IDUs for participation in the BTS. RDS is a validated probability sampling method devised for use with hidden populations such as sex workers, men who have sex with men, and IDUs, [11] that is based on social network theory and builds on conventional snowball sampling. RDS has previously been used to recruit participants in many HIV biological and behavioural surveillance studies [12]. It uses peer networks for recruitment of participants and involves payment of purposively recruited ‘seed’ participants, who then refer other participants. RDS data are analysed using RDSAT software that generates appropriately weighted estimated proportions with confidence intervals [11,13]. The weights are designed to account for patterns of recruitment. For this study, a sample size of 400 was estimated based on an ability to detect changes in proportions of 15% at follow-up surveys from estimated baseline values of 50% (which yield the biggest sample size), an alpha level of 0.05 for a two-sided test, and a power of 90%. A design effect of 1.5 was applied to account for intra-class correlation [14].

### Data collection

Participants responded to an extensive interviewer-administered questionnaire that was adapted from the one used in the previously mentioned IBBA study undertaken among high risk groups in India [7,15]. This questionnaire has been well-described elsewhere [16]. The adapted BTS questionnaire gathered information on socio-demographics, drug use and injecting practices, sexual behaviour and condom use, knowledge of HIV, and exposure to interventions. Field pre-testing of the questionnaire was undertaken prior to baseline measurement. Site supervisors, interviewers, coupon managers and screeners were provided with training to effectively undertake their respective roles.

To be eligible to participate in the study IDUs needed to be 18 years of age or older, and have injected drugs for non-medical purposes at least once within the last six months. Screeners with good knowledge of the local injecting drug use context were employed to ensure participants met the eligibility criteria. To start the RDS sampling chain, four seeds in each of the districts were purposively selected to reflect a diversity of demographic characteristics, particularly; age, drug use pattern, geographical location, marital status and employment status. Each seed was provided with three coded coupons to distribute to the participants they recruited, and each subsequent participant was also handed three coded coupons to distribute amongst their peers. This process continues in waves until the desired sample size is attained. This approach potentially results in recruitment of IDUs who attend Project ORCHID NGO services, those who attend non-Project ORCHID services, and those who do not use any services.

### Statistical analysis

Data were entered using CSPro Software and transferred to SPSS and Excel for cleaning and variable construction. Descriptive analysis was undertaken using RDSAT (version 6.0) to generate adjusted proportion estimates with 95% confidence intervals. Logistic regression analyses were conducted using SPSS version 18.0 to estimate the strength of association between exposure to interventions and needle sharing, condom usage and HIV testing; the data for this analysis are grouped at the state (rather than district) level. Odds ratios are presented with their respective 95% confidence intervals and are adjusted for the following variables; district, age, ever been married, literacy, age at initiation into injecting drug use, most commonly used drug, and frequency of drug use during the previous week. These adjusted odds ratios were derived from unweighted estimates. Unfortunately there is no consensus among statisticians as to whether data gathered through RDS can be appropriately weighted for multivariate analysis, and we encourage policy planners to interpret these regression findings with some caution [17].

## Results

### Participant characteristics

The desired minimum sample size of 400 was achieved through RDS in all four districts at both baseline and follow-up. Some of the participants recruited at follow-up reported participating in the baseline survey; 13.3% (n=56) in Ukhrul, 22.9% (n=95) in Chandel, 29.7% (n=127) in Kiphire, and 28.1% (n=126) in Zunheboto.

The characteristics of IDU participants did not change substantially between round 1 and round 2 except with respect to age (Table 1). The sample was predominantly under the age of 35 years in all four districts at both time points. At baseline and follow-up a large proportion of participants were under 25 years of age in Ukhrul (65.5% and 41.2%), Kiphire (84.1% and 61.0%), and Zunheboto (61.4% and 45.8%). However, the proportion of those aged under 25 years had fallen in these districts indicating a moderately older profile among the participants at follow-up.

**Table 1:** Socio-demographic profile

	Manipur				Nagaland			
	
	Ukhrul		Chandel		Kiphire		Zunheboto	
	
	(%)		(%)		(%)		(%)	
	2007	2009	2007	2009	2007	2009	2007	2009
**N ***(All respondents)*	421	421	420	415	417	427	422	449

**Age** *18-24* *25-34* *35*+	65.5 26.1 8.3	41.2 42.3 16.3	23.8 53.9 22.1	26.5 46.9 26.5	84.1 13.1 2.8	61.0 36.3 2.6	61.4 30.3 8.3	45.8 43.6 10.4

**Literacy** *Cannot read and write* *Can read only* *Can read and write*	0.2 1.9 97.7	6.4 3.2 90.3	25.9 2.2 71.7	6.1 1.7 88.6	34.0 4.3 61.6	12.8 9.0 78.1	24.7 3.3 71.9	19.3 13.1 67.5

**Occupation** *Unemployed* *Student* *Employed* *Other***^a^**	55.4 24 6.2 14.4	42.6 8.1 13.0 36.1	14.9 4.4 21.5 58.9	23.1 8.4 25.2 43.2	26.9 26.4 10.2 35.4	37.2 19.2 19.2 24.2	58.9 7.1 11.9 22.1	63.4 13.3 12.3 10.6

**Ever been married** *Yes* *No*	16.0 83.9	27.1 72.8	49.8 50.1	49.8 48.8	19.4 80.6	24.6 75.3	13.1 86.6	29.5 70.4

**Age at first injection** *Younger than 18* *18-24* *25-34* *35 or older*	23.3 68.7 7.3 0.6	27.8 69.1 1.2 2.1	3.4 49.2 36.7 10.8	2.4 49.3 35.8 12.5	19.6 75.8 4.6 0.0	8.7 75.0 16.3 0.0	13.8 64.3 19.0 2.9	1.2 70.5 25.3 3.0

**Two most commonly used drugs^b^** *Heroin* *SP*	77.0 77.3	86.1 25.9	97.9 9.2	96.2 8.9	11.1 99.2	44.4 98.4	-- --	8.6 97.0

**Ever had sexual intercourse** *Yes* *No*	81.1 18.5	89.9 10.1	75.0 24.9	83.8 16.1	82.7 17.3	86.6 13.3	71.5 28.4	90.2 9.7

**^a^** Other category includes agricultural, small time business, and unskilled labour.

**^b^** Multiple responses allowed.

-- Data unavailable for analysis.

Across all four districts a relatively small proportion of participants were employed, and the majority were literate and sexually experienced. In Ukhrul, Kiphire and Zunheboto most had been married, whereas in Chandel only approximately half had been married. The most commonly used drug in Manipur was heroin, whilst in Nagaland the most commonly used drug was Spasmoproxyvon. More than two-thirds reported first injecting drugs for non-medical purposes when they were under the age of 25 years in Ukhrul, Kiphire and Zunheboto; approximately half were under the age of 25 years in Chandel.

### HIV risk behaviours

#### Manipur

In Manipur (Table 2), the proportion of IDUs reporting that they injected at least once daily increased in Ukhrul (36.2% to 58.1%) and decreased in Chandel (96.1% to 75.6%). There was a large improvement in safe injecting practices in Ukhrul evidenced by a substantial increase in the proportion of IDUs that did not share needles with anyone during the past month (59.6% to 91.2%) or at the last injection (71.1% to 97.0%). Additionally, there was a decrease in the proportion of IDUs that drew up drug solutions from a common container at last injection (26.0% to 10.3%), or used to but had stopped (5.6% to 16.9%), between baseline and follow-up. In Chandel, very few IDUs reported sharing needles at last injection and during the past month at both baseline and follow-up, however, there was an increase in the proportion having drawn up drug solutions from common containers at last injection (6.6% to 24.6%). The majority of IDUs in Ukhrul and Chandel had between 1 and 3 female sexual partners in the 12 months preceding both baseline and follow-up questionnaires. The proportion of IDUs using condoms at last sex was higher with casual partners than with regular partners in both Manipur districts. There was little change in the proportion of IDUs using condoms at last sex, except in Ukhrul where a greater proportion using condoms with casual partners at follow-up (60.2% to 80.7%). The proportion of IDUs self-reporting STI symptoms decreased in Ukhrul (37.3% to 21.3%) and increased in Chandel (8.0% to 16.4%).

**Table 2:** Profile of injecting drug use and sexual practices

	Manipur			
	
	Ukhrul		Chandel	
	
	Baseline % (95% CI)	Follow-up % (95% CI)	Baseline % (95% CI)	Follow-up % (95% CI)
**Frequency of injecting in the last week** *At least once daily* *4-6 times a week* *2-3 times a week* *Only once* *Don’t know / remember*	36.2 (29.2 – 43.0) 17.7 (12.8 – 22.0) 34.0 (27.8 – 39.4) 10.9 (7.3 – 17.4) 1.2 (0.3 – 2.6)	58.1 (0.50 – 65.9) 10.7 (6.6 – 16.0) 18.2 (12.6 – 25.1) 12.6 (7.6 – 17.4) 0.3 (0.1 – 0.7)	96.1 (93.5 – 97.7) 0.7 (0.2 – 1.4) 1.5 (0.6 – 2.9) 1.8 (0.7 – 3.1) --	75.6 (70.1 – 81.0) 5.4 (3.4 – 7.7) 8.5 (5.4 – 12.0) 10.5 (6.4 – 14.3) --

**Did NOT share needles / syringes during past month**	59.6 (53.6 - 65.2)	91.2 (88.0 – 94.0)	96.3 (94.2 – 98.0)	95.3 (92.7 – 97.5)

**Did NOT share needles / syringes at last injection**	71.1 (66.4 – 76.0)	97.0 (95.1 – 98.2)	97.0 (95.4 – 98.4)	98.9 (97.9 – 99.7)

**Drew up drug solutions from a common container at last injection**	26.0 (22.1 – 30.9)	10.3 (6.9 – 13.9)	6.6 (4.5 – 8.8)	24.6 (19.1 – 29.9)

**Number of female partners had sex with in the past 12 months** *None* *1* *2-3* >*3*	3.8 (1.6 – 6.5) 34.4 (28.9 – 41.7) 37.3 (31.1 – 43.1) 24.5 (19.5 – 28.9)	12.7 (8.7 – 19.0) 40.3 (32.3 – 45.5) 22.5 (17.4 – 29.0) 24.5 (19.1 – 30.9)	9.6 (6.4 – 13.1) 73.6 (66.7 – 78.1) 11.6 (8.3 – 16.4) 5.2 (2.8 – 8.8)	20.3 (16.7 – 25.9) 67.0 (60.9 – 72.3) 11.6 (7.3 – 15.2) 1.1 (0.4 – 1.9)

**Condom use at last sex** *Regular partner* *Casual partner*	40.5 (29.0 – 44.2) 60.2 (55.9 – 72.2)	48.0 (36.4 – 57.4) 80.7 (67.9 – 87.1)	34.7 (23.5 – 45.7) 57.4 (37.3 – 77.1)	27.4 (18.6 – 34.2) 63.6 (51.5 – 85.6)

**STI symptoms during past 12 months**	37.3 (32.5 – 41.9)	21.3 (16.4 – 25.8)	8.0 (5.8 – 10.6)	16.4 (12.5 – 20.5)

	**Nagaland**			
	
	**Kiphire**		**Zunheboto**	
	
	Baseline % (95% CI)	Follow-up % (95% CI)	Baseline % (95% CI)	Follow-up % (95% CI)

**Frequency of injecting in the last week** *At least once daily* *4-6 times a week* *2-3 times a week* *Only once* *Don’t know / remember*	43.0 (38.5 – 51.9) 28.0 (22.1 – 32.8) 22.7 (17.3 – 26.4) 5.6 (2.4 – 8.4) --	28.8 (22.8 – 33.1) 24.6 (18.8 – 29.3) 33.7 (28.7 – 41.8) 12.9 (8.2 – 18.6) --	36.6 (29.3 – 43.4) 30.6 (24.1 – 36.2) 32.1 (26.4 – 40.2) 0.7 (0.0 – 1.7) --	26.7 (20.7 – 33.9) 52.0 (45.7 – 59.0) 13.6 (10.6 – 17.8) 7.0 (3.6 – 8.2) 0.7 (0.0 – 1.5)

**Did NOT share needles / syringes during past month**	85.4 (81.7 – 94.0)	64.1 (59.1 – 68.8)	68.7 (61.6 – 75.1)	72.0 (66.8 – 76.6)

**fDid NOT share needles / syringes at last injection**	63.3 (18.3 – 87.7)	68.8 (63.7 – 73.5)	45.5 (30.7 – 50.8)	73.8 (68.6 – 78.3)

**Drew up drug solutions from a common container at last injection**	30.7 (26.8 – 35.1)	46.8 (40.9 – 52.8)	41.3 (35.9 – 45.5)	53.8 (47.9 – 59.1)

**Number of female partners had sex with in the past 12 months** *None* *1* *2-3* >*3*	3.5 (1.9 – 6.0) 45.6 (39.9 – 51.3) 33.2 (27.3 – 38.0) 17.7 (14.6 – 21.3)	0.0 (0.0 – 0.0) 38.1 (24.9 – 36.6) 31.0 (24.9 – 36.6) 30.9 (25.4 – 37.0)	3.7 (1.8 – 5.1) 42.7 (32.8 – 47.1) 27.5 (22.3 – 33.4) 26.1 (23.7 – 34.6)	0.3 (0.0 – 0.06) 54.2 (50.1 – 61.8) 29.7 (24.7 – 36.1) 15.8 (10.2 – 17.3)

**Condom use at last sex** *Regular partner* *Casual partner*	47.3 (41.2 – 58.3) 44.6 (36.7 – 57.2)	38.1 (31.1 – 46.2) 73.6 (64.2 – 83.2)	52.4 (47.2 – 61.5) 44.9 (17.1 – 64.6)	33.4 (27.0 – 41.0) 86.0 (73.8 – 90.2)

**STI symptoms during past 12 months**	9.5 (7.3 – 12.5)	19.4 (15.5 – 23.4)	21.1 (17.2 – 26.0)	11.4 (8.7 – 14.8)

#### Nagaland

In Nagaland (Table 2), the proportion of IDUs injecting at least once daily decreased in Kiphire (43.0% to 28.8%) and Zunheboto (36.6% to 26.7%). Mixed results were obtained with respect to changes in the practice of sharing needles / syringes. In Zunheboto, the proportion of IDUs that did not share at the last injection increased substantially (45.5% to 73.8%), while the proportion sharing at least once during the past month remained largely unchanged (68.7% to 72.0%). In Kiphire, the proportion of IDUs that did not share needles in the past month decreased from 85.4% to 64.1%, whilst the proportion not sharing needles at the last injection remained largely unchanged (63.3% to 68.8%). There was a modest increase in the proportion drawing up drug solutions from common containers at last injection in both Kiphire (30.7% to 46.8%) and Zunheboto (41.3% to 53.8%). Very few IDUs in Kiphire and Zunheboto had no female sexual partners in the 12 months preceding both baseline and follow-up questionnaires; approximately one third had between 2 and 3 sexual partners. A greater proportion of IDUs used condoms with casual partners at last sex at follow-up in both Kiphire (44.6% to 73.6%) and Zunheboto (44.9% to 86.0%). However, there was a decrease in the proportion using condoms with regular partners at last sex in both Kiphire (47.3% to 38.1%) and Zunheboto (52.4% to 33.4%). The proportion of IDUs self-reporting STI symptoms decreased in Zunheboto (21.1% to 11.4%) and increased in Kiphire (9.5% to 19.4%).

### Exposure to interventions and knowledge of HIV

Table 3 presents indicators of exposure to interventions and awareness of HIV. The general overall trend was an increase in IDUs accessing NGO services and a greater awareness of HIV, although some district level variation was evident. The proportion of IDUs accessing NGO services during the previous 6 months increased substantially in Ukhrul (43.2% to 81.8%) and Kiphire (54.7% to 76.6%), remained high in Chandel (84.5% to 84.4%), and did not improve in Zunheboto (56.2% to 61.5%). There was an increase in IDUs registering with ORCHID NGOs in the two Manipuri states of Ukhrul (31.7% to 69.5%) and Chandel (26.3% to 70.9%), but minimal changes in the Nagaland districts of Kiphire (21.1% to 29.7%) and Zunheboto (48.5% to 39.8%). A larger proportion of IDUs reported usually obtaining needles from the needle and syringe program (NSP) at follow-up in both Ukhrul (27.4% to 67.6%) and Kiphire (38.0% to 71.0%), whilst the proportion remained relatively high in Chandel (86.5% to 68.0%), and there was little improvement in Zunheboto (36.5% to 35.9%). The proportion of IDUs who personally knew someone with HIV increased in Ukhrul (76.1% to 94.1%), Chandel (37.8% to 63.0%), and Kiphire (11.3% to 38.4%), and decreased in Zunheboto (23.3% to 6.0%). There was a reduction in the proportion of IDUs rating themselves at no risk of getting HIV infection in all four districts; Ukhrul (28.3% to 17.9%), Chandel (34.6% to 26.3%), Kiphire (31.0% to 4.9%) and Zunheboto 23.0% to 9.7%). A greater proportion of IDUs reported having had an HIV test at follow-up in Ukhrul (20.2% to 41.1%), Kiphire (10.6% to 52.0%) and Zunheboto (23.2% to 51.9%), and remained much the same in Chandel (41.9% to 45.8%).

**Table 3:** Exposure to interventions and awareness of HIV

	Manipur			
	
	Ukhrul		Chandel	
	
	Baseline % (95% CI)	Follow-up % (95% CI)	Baseline % (95% CI)	Follow-up % (95% CI)
**Registered with an ORCHID NGO**	31.7 (26.9 – 37.4)	69.5 (64.8 – 75.7)	26.3 (22.5 – 32.6)	70.9 (65.6 – 76.2)

**Accessed NGO services in last 6 months**	43.2 (37.9-49.3)	81.8 (76.3 – 87.2)	84.5 (80.7 – 88.7)	84.4 (79.8 – 88.8)

**Usually obtain needles from needle / syringe program**	27.4 (23.5 – 33.9)	67.6 (62.1 – 74.3)	86.5 (82.9 – 90.4)	68.0 (63.1 – 76.7)

**Personally know someone with HIV**	76.1 (71.8 – 80.3)	94.1 (91.9 – 97.6)	37.8 (33.0 – 44.8)	63.0 (57.7 – 68.0)

**Rate risk of HIV infection** High Moderate None Don’t know	24.9 (20.9 – 28.8) 10.8 (8.1 – 13.8) 16.9 (13.2 – 20.2) 28.3 (23.9 – 33.3) 18.6 (14.4 – 23.1)	37.8 (32.0 – 42.9) 15.9 (12.1 – 20.2) 24.4 (2.0 – 29.7) 17.9 (14.0 – 21.9) 4.0 (1.7 – 6.3)	6.0 (3.8 – 8.3) 10.6 – 7.6 – 13.1) 43.5 (38.4 – 48.8) 34.6 (29.5 – 39.4) 5.3 (3.3 – 8.3)	8.8 (6.2 – 12.0) 10.1 (7.1 – 12.7) 50.7 (45.4 – 55.8) 26.3 (21.9 – 31.7) 4.1 (1.9 – 6.8)

**Ever undergone HIV test**	20.2 (16.5 – 24.8)	41.1 (36.3 – 46.8)	41.9 (36.3 – 47.3)	45.8 (40.4 – 51.0)

	**Nagaland**			
	
	**Kiphire**		**Zunheboto**	
	
	Baseline % (95% CI)	Follow-up % (95% CI)	Baseline % (95% CI)	Follow-up % (95% CI)

**Registered with an ORCHID NGO**	21.1 (17.1 – 25.2)	29.7 (24.3 – 36.1)	48.5 (42.3 – 54.8)	39.8 (33.3 – 46.3)

**Accessed NGO services in last 6 months**	54.7 (48.9 – 60.9)	76.6 (71.9 – 81.1)	56.2 (50.4 – 63.0)	61.5 (56.5 – 69.3)

**Usually obtain needles from needle / syringe program**	38.0 (34.3 – 44.1)	71.0 (65.9 – 76.3)	36.5 (31.5 – 44.3)	35.9 (29.1 – 45.6)

**Personally know someone with HIV**	11.3 (8.9 – 14.7)	38.4 (33.1 – 43.2)	23.3 (19.2 – 28.7)	6.0 (3.9 – 8.6)

**Rate risk of HIV infection** High Moderate Low None Don’t know	17.4 (12.0 - 21.9) 22.4 (16.9 – 27.8) 11.4 (8.3 – 17.2) 31.0 (22.7 – 37.1) 17.8 (13.3 – 24.6)	12.1 (8.9 – 15.1) 32.1 (28.0 – 37.4) 43.9 (38.9 – 48.7) 4.9 (2.8 – 7.1) 5.8 (3.5 – 8.5)	2.5 (1.2 – 4.1) 8.6 (5.7 – 10.8) 53.7 (48.7 – 59.1) 23.0 (19.5 – 27.3) 12.2 (8.5 – 15.7)	12.0 (8.7 – 15.1) 26.4 (22.8 – 30.8) 39.9 (35.3 – 45.1) 9.7 (7.2 – 12.9) 10.2 (7.2 – 12.4)

**Ever undergone HIV test**	10.6 (5.9 – 13.5)	52.0 (46.5 – 56.7)	23.2 (19.2 – 28.3)	51.9 (46.1 – 57.0)

### Associations between exposure to interventions and HIV risk behaviours of IDUs

Analyses were conducted on follow-up data at state level to estimate the strength of association between exposure to interventions and: needle sharing, condom usage and HIV testing. Odds ratios presented in Table 4 were adjusted for district, age, having ever been married, literacy, age at initiation into injecting drug use, most commonly used drug, and frequency of drug use during the previous week.

**Table 4:** Association between programme exposure and HIV risk behaviours of IDUs at follow-up (2009)

	Adjusted odds ratios (95% CI)				
	
	Shared needles / syringes at last injection	Shared needles / syringes during the past month	**Condom last sex (regular partner)**^a^	**Condom last sex (casual partner)**^b^	HIV test
	
	Manipur				
**Accessed NGO services in last 6 months**	0.51 (0.14 - 1.90)	0.35** (0.17 - 0.74)	3.00** (1.45 - 6.22)	1.19 (0.44 - 3.26)	1.52 (0.88 - 2.63)

**Registered with an ORCHID NGO**	0.47 (0.16 - 1.29)	0.59 (0.32 - 1.08)	2.75*** (1.63 - 4.64)	1.49 (0.78 - 3.00)	1.49* (1.01 - 2.19)

**Usually obtain needles from NSP**	0.42 (0.15 - 1.17)	0.32*** (0.18 - 0.57)	2.94*** (1.77 - 4.91)	0.66 (0.32 - 1.36)	1.62* (1.10 - 2.39)

	**Nagaland**				

**Accessed NGO services in last 6 months**	0.53** (0.35 - 0.79)	0.78 (0.52 - 1.15)	1.15 (0.69 - 1.92)	3.98** (1.80 - 8.76)	1.27 (0.87 - 1.85)

**Registered with an ORCHID NGO**	0.57** (0.41 - 0.87)	0. 63* (0.44 - 0.90)	1.87* (1.13 – 3.07)	1.61 (0.86 – 2.98)	0.89 (0.64 - 1.25)

**Usually obtain needles from NSP**	0.67* (0.46 - 0.98)	0.96 (0.68 - 1.42)	1.35 (0.83 - 2.19)	2.75** (1.35 – 6.00)	0.77 (0.55 - 1.10)

^a^ Only IDUs who had a regular partner were included in this analysis; 422 in Manipur, 486 in Nagaland.

^b^ Only IDUs who had a casual partner were included in this analysis; 246 in Manipur, 313 in Nagaland.

* p<0.05, ** p<0.01, *** p<0.001.

Note: Odds ratios were adjusted for the following variables; district, age, ever been married, literacy, age at initiation into injecting drug use, most commonly used drug, and frequency of drug use during the previous week.

Evidence supporting an association between program exposure and: reduced needle sharing, increased condom use, and increased HIV testing emerged.

In Nagaland, registration with an ORCHID NGO was significantly associated (p<0.05) with: lower odds of sharing needles during the previous month (OR=0.63) and at the last injection (OR=0.57); and higher odds of condom use during the last sexual encounter with regular partners (OR=1.87). Similarly, accessing NGO services in the last six months and usually obtaining needles from the needle and syringe program was associated with: reduced odds of sharing needles at the last injection (OR=0.53 and 0.67 respectively); and higher odds of using a condom at last sex with a casual partner (OR=3.98 and 2.75 respectively). Participation in HIV testing was unaffected by program exposure in Nagaland.

In Manipur, registration with an ORCHID NGO was significantly associated with higher odds of condom use during the last sexual encounter with a regular partner (OR=2.75). Having accessed NGO services in the last 6 months was associated with: lower odds of sharing needles during the previous month (OR=0.35); and higher odds of condom use at last sex with a regular partner (OR=3.00). Additionally, usually obtaining needles from the needle syringe program was associated with: lower odds of sharing needles during the past month (OR=0.32); and higher odds of condom use for last sex with a regular partner (2.94). In Manipur, the odds of having had an HIV test increased for those IDUs registered with an ORCHID NGO (OR=1.49) and those usually obtaining needles from the needle syringe program (OR=1.62).

## Discussion

While the findings from this study present a mixed picture in relation to changes in HIV risk behaviours and HIV prevention service usage among IDUs in two districts of Manipur and two districts of Nagaland, the overall trend is a positive one. Consistent associations between exposure to NGO HIV prevention services, including Project ORCHID services, and reduced risk of engagement in HIV risk behaviours are clearly evident. Additionally, exposure to interventions was associated with increased chance of HIV testing in Manipur.

### Profile of injecting drug users

An important aspect of the demographic profile is that a substantial proportion of IDUs in all four districts were less than 25 years of age, comparable to IBBA findings in four other districts of Manipur and Nagaland [7]. Additionally, the majority of IDUs first injected drugs before the age of 25 years in Ukhrul, Kiphire and Zunheboto; in Chandel approximately half of IDUs had first injected at this young age. Many of these men are entering into injecting drug use during their adolescent years, and studies have demonstrated that younger age and earlier age of initiation into injecting are associated with HIV infection [18-21]. This raises policy and programming challenges, some of which may be sensitive in this very conservative context. Adopting a youth and equity lens [22,23] is important to ensure: 1) adolescents have access to HIV prevention resources (i.e. condoms, needles, etc), 2) that drug services and sexual and reproductive health services are “youth-friendly” i.e. they engage with and respond to the information and treatment needs of young people, 3) that information and education on drug use and sexual and reproductive health is widely available to adolescents, and 4) that opportunities to interrupt the transition to injecting drug use (e.g. life skills programs) are explored through further research and programming.

### HIV prevention service usage

A recent (2009) document produced by a collaboration between WHO, UNODC and UNAIDS [24], identified a range of coverage targets and impact indicators for HIV prevention programs working with IDUs. One coverage indicator is the percentage of IDUs regularly reached by NSPs. Coverage is judged to be high if >60% of IDUs are regularly being reached by NSPs. On the basis of this indicator, the IDUs in Ukhrul, Chandel and Kiphire were all receiving high coverage by 2009. Even though the proportion of IDUs attending HIV prevention services generally increased between the two BTS rounds, some points of concern were noted and possibly warrant programmatic adjustment, including a decrease in the proportion of IDUs obtaining needles from NSPs in Chandel, and consistently poor uptake of services in Zunheboto.

IDU participation in HIV testing is an important part of HIV prevention. The recommended coverage target for HIV testing among IDUs is 75% of IDUs each year [24]. Despite marked increases in the proportion of IDUs who had been HIV tested between the two BTS rounds, the IDUs in Manipur and Nagaland are a long way from achieving the recommended coverage target for HIV testing. At follow-up, only around half reported ever having had an HIV test. The poor uptake of HIV testing among IDUs in these states was also reported in the IBBA study [7], and is the result of a constellation of factors including poor health system infrastructure to support HIV testing, geographically remote communities, and lack of perceived risk. In Chandel in 2009, 77% of IDUs judged themselves to be at no or low risk of HIV, as did 42% in Ukhrul, 49% in Kiphire and 49% in Zunheboto. More research is required to fully understand why IDUs in Manipur and Nagaland do not participate in HIV testing, so that programs can be designed to encourage better uptake.

### Changes in injecting behaviours of IDUs

High risk injecting behaviours were common at baseline in Ukhrul, Kiphire, and Zunheboto, with approximately 30-50% of IDUs sharing needles at last injection. Risky injecting behaviour has been found to be common among IDUs in other districts of Manipur and Nagaland [7] and elsewhere in India [25]. One of the impact indicators identified in the WHO/UNODC/UNAIDS document is an increase in the percentage of IDUs reporting use of sterile injecting equipment the last time they injected [24]. Based on the responses to the BTS question about not sharing needles and syringes at the time of last injection (a roughly equivalent indicator), an increase to almost 100% was reported in Ukhrul and a similarly high level of safe injecting was maintained in Chandel. A substantial increase in the percentage of IDUs (from 45% to 74%) reported not sharing at the time of last injection in Zunheboto, but in Kiphire there was no real improvement in the percentage not sharing with their last injection – where one third of IDUs consistently reported sharing at their last injection, and the proportion sharing in the last month had increased over time.

The proportion of IDUs drawing up drug solutions from common containers at the time of their last injection is a major cause for concern as this is an effective route of transmission for hepatitis C virus [31]. In three of the four districts the proportion reporting this practice increased between the two BTS rounds (Ukhrul was the exception), and between 10-54% of IDUs, depending on district, reported doing this in 2009. This is particularly concerning given the background prevalence of hepatitis C infection in Manipur in particular, which was 56% in Bishnupur and 78% in Churachandpur districts in 2006 [7]. In a development context where treatment of hepatitis C is not supported in the same way that ART is, and most IDUs could never afford to pay for the treatment, hepatitis C prevention is at least as important as HIV prevention.

Sharing injecting equipment is associated with a number of factors including the type of drug being injected, place of injection, availability of clean injecting equipment and awareness of the risks of sharing. Rhodes et al [29] apply a broader understanding of risk to examine the social structural production of HIV risk behaviour, identifying the inseparability of micro, meso and macro-level factors that shape the environment in which risk is produced beyond a focus on the individual. Under this paradigm risk environments are shaped by factors external to the individual such as trade, migration, neighbourhood disadvantage and transition, injecting environments, the criminal justice system, social norms and networks, social capital, law enforcement, and armed conflict.

A recent study among HIV positive IDUs in Manipur identified some important local socio-structural influences on injecting risk behaviours including inadequate coverage of needle / syringe programs (including in prisons), limited access to pharmacy-sold equipment, IDUs avoiding carrying clean injecting equipment due to fear of harassment by police and anti-drug groups, and withdrawal symptoms superseding health concerns [30]. A number of other studies have highlighted the ways in which social networks influence the extent to which IDUs engage in risky injecting behaviours. IDUs are more inclined to share injecting equipment when they are a member of a social network that is large, longstanding and dense (meaning many ties between network members), and when sharing injecting equipment is normative and an expression of social bonding [26,27]. These are features of many IDU groupings in Manipur and Nagaland [28]. Further research is required to better understand the broader socio-structural drivers of HIV risk in Manipur and Nagaland to support interventions that create local environments that support individual, group and community-level changes.

### Changes in sexual behaviours of IDUs

Reaching IDUs with safe sex messages is in many ways a greater challenge for harm reduction programs than promoting safe injecting behaviours. The WHO / UNODC / UNAIDS document indentified an increase in the percentage of IDUs reporting the use of a condom the last time they had sex as an impact indicator [24]. For the IDUs in this study there was overall no real increase in the proportion reporting condom use during the last sexual encounter with regular partners, but an increase in the proportion using condoms in the last sexual encounter with casual partners was evident in all districts except Chandel. Programmatic focus on the prevention of sexual transmission of HIV in drug use settings is important [32]. This is particularly the case in Nagaland where around half of the IDUs had more than one sexual partner in the last year, and where the 2006 IBBA study highlighted a high syphilis prevalence among IDUs in Nagaland (7% and 19% in Phek and Wokha districts respectively) [7], indicating sexual risk taking and possible co-infection of HIV and an ulcerative STI, increasing the risk of HIV transmission to sexual partners. Further research and analysis of behavioural data is required to more comprehensively understand the sexual behaviours of IDUs in Northeast India and to better appreciate the sex-related risks of HIV transmission among this group.

### Association between exposure to HIV prevention services and HIV risk behaviours

Our state-level analysis found consistent associations between exposure to HIV prevention services and a reduced likelihood of sharing needles and an increased likelihood of using condoms. Exposure to HIV prevention services was also associated with increased likelihood of participation in HIV testing in Manipur; there was no similar association in Nagaland. The strength of the full impact of the HIV prevention services on HIV risk behaviours is difficult to gauge due to the nature of the cross-sectional study design, but in our analysis it appears to be substantial. In Nagaland the likelihood of sharing needles over the past month was reduced by approximately 40% among IDUs who were registered with an ORCHID NGO. In Manipur, IDUs who had NOT accessed an NGO service during the past 6 months were almost three times more likely to have shared a needle during the past month. The likelihood of using condoms with regular partners during the last sexual intercourse was tripled among IDUs in Manipur who had accessed NGO services during the past 6 months, and nearly doubled among IDUs in Nagaland who were registered with an ORCHID NGO. These results provide evidence of an association between exposure to HIV prevention services and a reduced likelihood of engaging in HIV risk behaviours.

### Inter and intra-state variations

The findings from this BTS study varied considerably between states, and between districts within states. The patterning of HIV risk behaviours and HIV prevention service usage in this complex setting is influenced by a constellation of factors including the supply and types of drugs used, awareness of risks associated with sharing injecting equipment, the social norms among local networks of IDUs in relation to sharing, the effectiveness of the NGOs offering the programs, the availability of clean needles and syringes, the freedom to access the NSPs without harassment from police or pressure groups, geographical accessibility of the NSPs, and the extent of empowerment among IDU networks.

The differences between the two states are not surprising as the pattern of drug use, and the maturity of the HIV epidemic and the response to it, are very different when the two states are compared. Manipur IDUs inject mainly heroin, while the Nagaland IDUs inject mainly pharmaceuticals (especially Spasmoproxyvon or SP). SP is often taken orally, and it is not uncommon for SP users to move in and out of injecting depending on the availability of the drug i.e. they are more likely to inject at times of drug shortage (but when in an injecting phase, SP users may inject up to 10 times/day). Additionally, SP is very injurious to veins, so it is difficult to inject every day for sustained periods of time. In contrast, dependant heroin users are much more likely to consistently inject every day over extended periods, even decades. As the HIV epidemic in Manipur is more severe and long-standing compared with Nagaland, the Manipuri NGOs are more experienced in delivering programs, and awareness of HIV is greater. Communities are more geographically isolated in Nagaland, and the political situation is more unstable in Manipur – and both of these factors interfere with the delivery of HIV prevention services. Similar types of variation are evident between districts within states as well. This sort of complexity contributes to the variability in outcomes observed in this study, and highlights the importance of NGO capacity to monitor what is happening locally, and to adjust the program accordingly.

### Limitations

These study findings are subject to several limitations. The use of RDS methods to recruit participants creates limitations for multivariate analysis; guidelines for multivariate analysis of RDS data are still under development and require validation. Despite this limitation, RDS results in probability based estimates of various participant characteristics. Reporting of certain behaviours may have been influenced by recall and social desirability bias with the result that socially unacceptable behaviours may have been under-reported. As with all cross-sectional study designs causal relationships can’t be firmly determined due to the difficulty in determining the temporal relationship between the predictors and the outcomes. With this study design it is hard to confirm that exposure to services causes a change in risk behaviours among IDUs; it is possible that the types of IDUs who attend services are less likely to engage in risk behaviours. However, the multivariate analysis in this paper highlights consistent associations between exposure to NGO HIV prevention services, including Project ORCHID services, and reduced risk of engagement in HIV risk behaviours. It is important to note that socio-structural factors influence HIV risk behaviours and these have not been reported on in this paper; for example, place of injection, access to clean injecting equipment, harassment by policy and anti-drug groups, incarceration, and poverty. Further analysis of the BTS and other data sources is required to comprehensively describe the socio-structural drivers of risk behaviours.

## Conclusion

Despite district-level differences, the results from this BTS study indicate that exposure to HIV prevention services, predominately delivered in this region by NGOs, is associated with a reduced likelihood of engaging in HIV risk behaviours. IDUs using HIV prevention services are more likely to engage in safe injecting and sexual practices, and effort is required to sustain / increase opportunities for IDUs to access these services. These outcomes are a noteworthy achievement in a very challenging context. Programmatic adjustments are required: 1) to further increase participation in HIV testing, 2) to deliver interventions that target the risk of sexual transmission of HIV and STIs, and 3) to improve awareness of the risk of Hepatitis C transmission associated with drawing drug solutions from common containers.

## Competing Interests

The authors declare that they have no competing interests.

## Authors’ contributions

All authors contributed to interpretation of the findings and development of the manuscript. GA and MK undertook the statistical analysis and wrote the first draft of the manuscript. CH coordinated the collection of data. All authors read and approved the final manuscript.
